# Community Power and Global Learning: *A Case Study of the Four Pillar Model of CORD from India to Michigan*

**DOI:** 10.5334/aogh.5043

**Published:** 2026-04-17

**Authors:** Vidya Kumar Ramanathan, Alexander Plum, Narender Paul, Kathleen Babuska, Morgan Pigott, Kshama Metre

**Affiliations:** 1 CORDUSA ‑ Global Network, Washtenaw County, Michigan, USA; 2 Corner Health Center, Ypsilanti, Michigan, USA; 3 CORD, Himachal Pradesh, India

**Keywords:** development, sustainability, women

## Abstract

A bidirectional, community‑based intervention in Washtenaw County, Michigan for women aged 12+, experiencing personal and systemic barriers, was developed based on learning from Himachal Pradesh, India. The partners, a global NGO, a local adolescent healthcare organization, and a community development network, leveraged community resources to help women achieve discrete goals in three arenas (access to healthcare, education for their children, and employment) by adapting and applying four “pillars” of the global NGO (participation, integration, sustainability, and networking) in maternal‑infant health care delivery and community engagement. The result was women reporting increased hopefulness, better health, and greater sense of agency in their lives, with implications for future work in improving health indicators and maternal‑infant health outcomes.

## Background

In the US, the concept of health equity programs centering community as the sole executor is relatively new and variable in implementation. The National Academy of Medicine first mentioned this in 2017 [1]. Community‑based Participatory Research has mid‑20th‑century roots but found traction in the public health space in the 1990s–2000s [2], with recent iterations promoting community‑led research [2] and expanded interpretation [3]. Participatory Action Research, which co‑emerged [4], empowers people to improve their own health [4]. Yet, most current social service and health equity programs are externally driven. The Robert Wood Johnson Foundation, however, champions Community Power. From 2020 to 2024, their Global Ideas for US Solutions Team reviewed Community Power’s global effectiveness, policy impacts, and challenges [5], finding community‑led programs more sustainable.

CORD, the Chinmaya Organisation for Rural Development, has promoted community‑led health equity for over 40 years in Himachal Pradesh and across rural India. CORD began in 1985 when Swami Chinmayananda, founder of Chinmaya Mission, saw that the mountainous Himachal terrain caused barriers to healthcare. CORD was thus founded as the Chinmaya Rural Primary Health Care and Training Center. Dr. Kshama Metre, a pediatrician and the National Director since its inception, initially developed the program to train village health workers and traditional birth attendants while providing primary care services to remote regions. She soon found that well‑being involves more than healthcare. The program has since evolved into what she calls sustainable integrated development. That is, it identifies and addresses determinants of health including access to care, rural development, and comprehensive social support, via a model that relies on self‑empowerment creating sustainable change. The Four Pillar Model of CORD, the program’s framework, focuses on community power.

Over these 40 years, CORD has developed satellites across India and now Sri Lanka. Core to most of these are Mahila Mandals, or women’s groups, centering women as active participants in development. These women’s groups address the whole family/community, working on poverty, health, disability, education, justice, environment, and self‑governance. Out of these have grown programs including physical and occupational therapy, domestic violence support, organic farming, men’s and adolescent groups, microcredit groups, and income‑generation programs, overall impacting 815,000 people just last year in India. Though difficult to quantify, it is estimated that over 6 million people have benefited since 1985 due to the women’s government involvement [6].

In 2009, then Head of Chinmaya Mission Swami Tejomayananda formed a sister organization in the US called CORDUSA, to support CORD India. CORDUSA also engages in social welfare programs. Swami Swaroopananda, Global Head of Chinmaya Mission now, started CORDUSA – Global Network in 2022 with a vision to bring sustainable integrated development, via the Four Pillar Model of CORD, to communities around the world. While Chinmaya Mission is a spiritual organization now in its 75th year, CORDUSA and CORDUSA–Global Network (CGN) are secular. As Chinmaya Mission has over 350 centers worldwide, the grassroots infrastructure is in place to create large‑scale sustainable impact. CGN is already starting work at about 20 sites outside of India/Sri Lanka, across the US, Kenya, and the UK, but there is great scope for growth.

In May 2022, CGN started with a pilot program in Ann Arbor, Michigan, and has since grown, following the Four Pillar Model. In Washtenaw County, Michigan, CGN collaborates with Community Action Network (CAN), a community organization with seven branches supporting under‑resourced families, allowing CGN access with accountability and trust. The participants in two neighborhoods hold monthly women’s groups, like Mahila Mandals, setting their own agendas. They discuss a range of topics including education, racism, medication pricing, housing, mental health, taxes, and domestic violence. Once, we had a history lesson: Black women who lived through Jim Crow and Indians through Partition, each shared the vital legacy of how that informs life now. CGN volunteers started by providing skill‑based support. Over time, this became unnecessary, as the neighborhood women built up capacity to identify and address their community’s needs, achieving sustainability. Community members are our partners rather than beneficiaries, so they become leaders with a rippling chain reaction of community self‑reliance.

Our pilot program is in Washtenaw County. Statistics from 2022 paint a picture of pronounced racial and socioeconomic disparities in health outcomes in this county—some of the starkest in Michigan. Women’s healthcare coverage and mental health is generally better than the state average, with only 4.9% of women lacking health insurance [7]. Still, significant health inequities persist, with a pronounced Black‑to‑White gap: Black infant mortality is 3.5x higher than among whites, wider than the statewide gap [8], without a clear cause [9]. Compared across insurance types, income‑constrained Medicaid recipients perform worse across all birth‑related metrics. In Washtenaw County, most Black women are either uninsured or on Medicaid; they are the only racial group who so disproportionately rely on this safety net [8].

**Figure 1:**
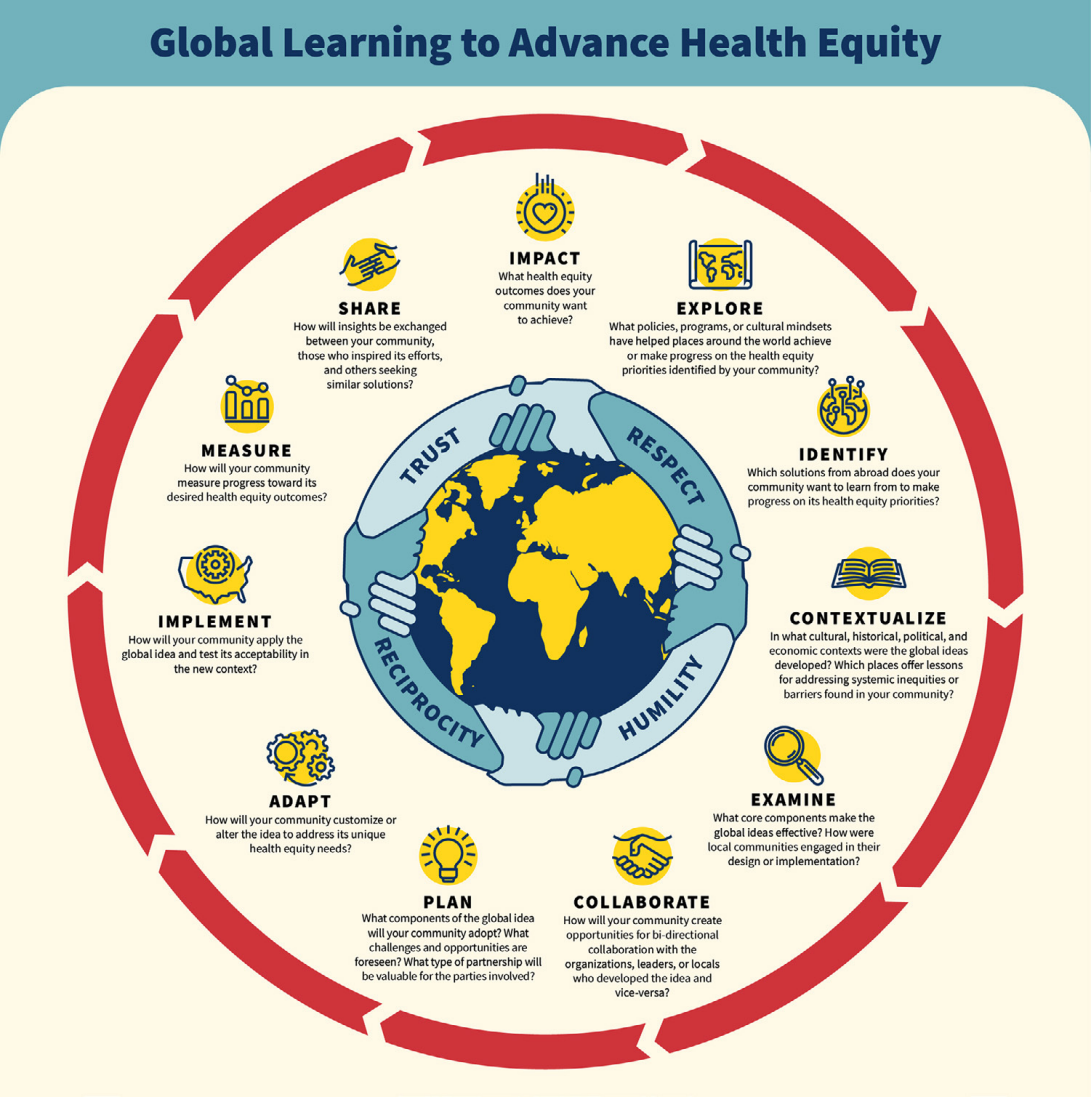
Global Learning to Advance Health Equity Framework, Global Learning for Health Equity Network.

To address these disparities via evidence‑based peer support groups [10] targeting social determinants of health [11], CORDUSA–Global Network, in collaboration with the Corner Health Center (CHC) was awarded funding in 2023 from the Global Learning for Health Equity (GL4HE) Network through an RWJF grant. While there are numerous global learning frameworks available, there has been a gap in data and implementation [12]. We used the Global Learning to Advance Health Equity Framework per the grant (Figure 1). While continuing to implement the Four Pillar Model of CORD with CGN’s existing community partners in Washtenaw County, CHC observed, learned, and participated as it implemented this model in its maternal infant health program. The overarching task was to see to what extent the Four Pillar Model could be extrapolated and applied to these new settings outside of India and what would be the impact. Please see Table 4 for an alphabetized index of abbreviations.

## The Four Pillar Model of CORD

The Four Pillar Model of CORD was developed by Dr. Kshama Metre (Table 1) [6].

**Table 1:** The Four Pillar Model of CORD.

1. **Participation**	The people in the communities develop and drive the programs. This means that programs are not developed in academia, at Board meetings, in think tanks, or even via focus groups. The people in the communities identify their unique needs, and together, they come up with solutions for their concerns.
2. **Integration**	Interventions are not single‑pronged but integrated across sectors. People need solutions to their food insecurity in conjunction with housing insecurity, access to care, immigration status, etc.
3. **Networking**	We live in societies with many resources. CORD’s philosophy is that by networking with organizations, people, and services already in place, we can be more effective and fill in gaps where they exist.
4. **Sustainability**	When the first three pillars are fulfilled with capacity building for communities as program drivers, holistic programming, and working with community networks, then programs automatically become sustainable long‑term.

## Evaluation Methodology for Individual Outcomes

### CORDUSA – global network at community action network’s bryant community center

This project and assessments were community‑driven. Outcomes were obtained as qualitative self‑reports over the last three years. Reports are in the form of written notes, videotaped discussions, and interviews between community members and neighbors (Table 2) after obtaining informed consent for each forum. As an internal program evaluation project, this was exempt from Institutional Review Board review.

**Table 2:** July 11, 2025—Notes on current concerns from discussions between neighbors of participants, CORDUSA – Global Network at Community Action Network’s Bryant Community Center.

TOPIC	NOTES
Food Insecurity	Food distribution centers closing
Access to Healthcare	Losing benefits Medicaid/Medicare cuts Loss of access to vaccines High cost of medications
Domestic Violence	“System doesn’t help women who are targeted” Concern for security
Immigration	Concern about ICE raids/family
Racism	Affects all interactions in all parts of society Concerns about quality of care Concerns about safety of Black and Brown children Microaggressions at work/school
Access to Education	Cutting of student loans “Children with special needs will not get the attention they need at school with this administration” Good education is required for employment, but pipeline is at risk
Elder Care	Difficulty getting social security Difficulty getting quality care Society not geared for elder care
Women’s Health	Access to care under great threat
Access to Housing	Increased rental rates for all, especially for seniors Lack of housing stability for lower‑income people
Financial Issues	401K at risk Unsure how to save money in this economy Concern about job security

### The corner health center

As a participant of GL4HE Network testing elements of its Framework (Figure 1), CHC sought to trial the “identification” and “exploration” phases in partnership with CGN. Both organizations’ participation evolved beyond these two introductory process phases into implementation of pilot projects, informed by a site visit to Himachal Pradesh to see the Four Pillar Model in action through women’s groups. Inspired by the visit, CHC desired to bring an adaptation of women’s groups to its flagship Ypsilanti location and apply the Four Pillar Model. From there, program sustainability and integration with other support services were realized.

From the outset, CHC hypothesized that the Four Pillar Model of CORD exemplified its mission. Guided by the Four Pillars, CHC listened to women patients to understand their needs by undertaking focus group discussions. Although staff‑initiated, the focus groups used a semi‑structured interview guide, found to help facilitate direction‑setting and ownership on the part of participants [13]. Recruitment took place onsite at CHC through word‑of‑mouth and flyers. When perinatal patients checked‑in, they were provided information about the study. Both methods directed prospective participants to a member of the maternal infant health program (MIHP) team to explain the study, answer questions, obtain consent, and collect contact information. As a clinical quality improvement project, although the study was exempt from Institutional Review Board review, ethical research procedures were closely followed.

More than 200 patients were solicited for feedback based on tracking, appointment data, and word‑of‑mouth count. Twenty expressed interest and were invited to participate. Due to various conflicts, seven participated in a semi‑structured 90‑minute focus group discussion in April 2024. Informed consent was obtained before the focus group. A script was read aloud by a research team member explaining the study purpose, its voluntary nature, risks and benefits, and obtaining verbal consent to record. Consent was documented on the audio recording, which, with the transcript, was saved in a password‑protected folder on a CHC‑owned, locked laptop, on a local drive only accessible by the research team. Discussions focused on CHC’s MIHP and its effectiveness at meeting community needs.

After the completion of the focus group, a verbatim transcript was created with identifying information removed. Thematic analysis was conducted, with both deductive codes (e.g. perinatal appointments, MIHP services) and inductive codes (e.g. informal support, childcare) identified and applied. The primary themes involved the need for more informal, supportive meetings. One participant remarked that “being pregnant is lonely.” This sentiment was shared among over half of the participants. Specific feedback on group requirements was recorded, with considerations like childcare and transportation emerging. “The biggest need is for someone to help watch my other kids,” remarked a participant, sharing a common perspective. An emphasis on patient autonomy around program topics was clear; “we know what we know, and what we don’t know yet.” It also spoke to the inherent sustainability potential of the groups. Limitations of the study include the purposive nature of recruitment and potential sample bias including homogenous demographics (i.e. over 80% of participants identified as Black women; all participants were enrolled in Medicaid). These limitations notwithstanding, the study faithfully represented the feelings and perspectives of participants.

Following the study, staff from CHC’s MIHP collaborated with its patients, health education team, and the county’s intermediate school district. Over 100 patients were requested to provide survey feedback on resulting program design changes. Follow‑up conversations with eight survey respondents were incorporated into the program design and decision to utilize Parent Cafés, a model of informal gatherings for patients with children who receive care at CHC. Participants of Parent Cafés have been found to “report a significant increase in their ability to learn communication and coping skills, develop their social support systems, and increase their parenting knowledge” [14].

The Parent Cafés were facilitated jointly by CHC staff and the intermediate school district’s “Success by 6” initiative, which provided in‑kind grant funding to supply trusted childcare and parent advisers monthly for six months. Following focus group feedback, Parent Café meetings followed no structured curriculum but started with dinner and interaction time. Staff were available to provide supportive information while participants set their own agendas. After six months, the Parent Cafés have continued as a core funded program. A more rigorous evaluation of the impact on patient outcomes will be held after 12 months of Parent Cafés, and statistical significance can be achieved.

When evaluated against the Four Pillars, it is clear how each element is reflected in CHC’s efforts. Participation by parent‑patients is at the center of each activity, from focus groups to Parent Cafés, agendas, and content. Integration has been achieved as Parent Cafés are nested within MIHP’s core offerings. Parent Cafés and CHC’s perinatal program function in the broader network of holistic care offered by partners in the region. Finally, sustainability was achieved through partnership with community organizations who shared a complementary mission and by CHC staff whose health education roles were perfectly suited to deliver the program.

## Outcomes for CORDUSA – Global Network at Community Action Network

### Individual‑level outcomes

Participants developed an increased understanding of their community’s needs. Women reported a greater sense of agency, trust, comfort, and support in their neighborhood. They stated that they feel loved in the meetings and feel hopeful about being able to create meaningful change in their lives.

### Organizational‑level outcomes

We developed a partnership between CGN and CAN at two neighborhoods in Washtenaw County, where an adaptation of the Four Pillar Model of CORD is centering women’s autonomy and fulfilling all four pillars with active participation, integration, and networking, resulting in sustainability.

CGN also partners with other organizations (Table 3) to help fulfill the women’s goals. This falls under the “Networking” Pillar of the Model, showcasing the power of community connections.

**Table 3:** Other local community partners.

**Tappan Middle School** – helps with navigating educational challenges. The principal of the school held a CGN workshop wherein she addressed individual needs of the women such as language barriers, technology questions, and post‑COVID learning difficulties, and has been an active participant in our group.
**Safe House** – Domestic Violence Shelter – *helps with education and resources re: domestic violence. After several workshops with Safe House, women expressed more comfort in reaching out for services and a greater understanding of available resources*.
**Washtenaw County Health Department** – *helps with healthcare resources. We have distributed pamphlets from the Health Department and noted an increased participation in Health Department programs and support groups by our members*.
**Washtenaw County Sheriff Department** – *helps with security resources and community engagement. The women asked for the Sheriff Dept to help them learn how to address their safety needs and expressed an increased sense of security after their educational sessions*.
**Briarwood Mall Community Outreach** – *helps connect more women to each other and other community organizations. The local mall holds community outreach events and includes CORDUSA – Global Network as a community partner to network with more local organizations*.

We have used a global learning framework (the Four Pillar Model of CORD) to create a comprehensive community development program, addressing such needs as housing, education, health, and safety, which all interplay in individual and community wellness. Seeing its budding success over these last three years has inspired the replication of this model at other centers in Michigan, across the US, Kenya, and the UK.

## Outcomes for the Corner Health Center

Since starting this project at CHC, the Parent Café model has gone from a six‑month trial to an integrated, monthly program offering for parent‑patients and their children. Initial findings confirm extant research on Parent Cafés. Participants report high satisfaction with having unstructured time alongside other mothers to network and unwind. Childcare, food, and health education were valued to create a supportive environment that allowed women to set their own direction, recognizing their individual expertise.

CHC used the GL4HE framework to identify a global learning partner, explore relevance and fit vis‑à‑vis local health challenges, and guide planning and decision‑making processes on the design and implementation of the health equity program. Staff expressed an increase in their knowledge and appreciation for global learning as a method by which health equity can be achieved locally, as well as admiration for the similarity in values between the Four Pillars and their own innate processes. On an organizational level, participation led CHC to expand its perinatal services with another project for women centering autonomy by ensuring Parent Cafés will continue as a core program.

## Impact on Health Equity

Through these Four Pillars‑inspired programs, people identify their own health disparities and then collaborate to find solutions for such systemic issues as racism in medicine, medication costs, and medical language barriers. They inspire one another and build a shared sense of community as they seek to thrive.

## Global Perspective

Appreciating the global perspective may have been the most significant value of participating in the GL4HE Network. To truly prioritize the values of community, humility, and reciprocity was eye‑opening. Putting community at the center of health equity work in the US is essential learning.

A site visit to Himachal Pradesh and learning directly from originators of the model inspired us, drove home important lessons, and activated our interest in bringing this work back to the US. Appreciating global health as not just international health, but a series of universal values that prioritize dignity, equity, justice, and worth has been transformative for all involved, and this worldview lends important perspective.

## Future Directions: Sustainability on a Larger Scale

Seeing is believing. Observing rural Mahila Mandal women identify systemic injustices and collaborate to resolve them such that active participation becomes the cultural norm is experiential learning.

Our goal is to scale up and roll out the Four Pillar Model of CORD at centers across the US and globally with close mentorship and continued experiential learning. Our next concurrent step is better documentation of processes and outcomes to foster replication across sites as we grow in scope and depth.

**Table 4:** Alphabetized index.

CAN – Community Action Network
CHC – Corner Health Center
CGN – CORDUSA – Global Network
CORD – Chinmaya Organisation for Rural Development
GL4HE – Global Learning for Health Equity
MIHP – Maternal Infant Health Program
RWJF – Robert Wood Johnson Foundation

Over the last 40 years in Himachal Pradesh, we have seen that women are the core of communities and often think beyond themselves. When women unite and understand the power of their own words and ideas, they go from inspired individuals to true change agents.

## References

[r1] National Academy of Medicine. Communities driving health equity. 2017. Accessed December 2025. https://nam.edu/our-work/programs/culture-of-health-program/communities-driving-health-equity/.

[r2] Wallerstein NB, Duran B. Using community‑based participatory research to address health disparities. Health Promot Pract. 2006;7(3):312–323. Accessed December 2025. https://journals.sagepub.com/doi/10.1177/1524839906289376. 16760238 10.1177/1524839906289376

[r3] Wallerstein N. Engage for equity: Advancing the fields of community‑based participatory research and community‑engaged research in community psychology and the social sciences. Am J Community Psychol. 2021;67(3–4):251–255. Accessed December 2025. https://pubmed.ncbi.nlm.nih.gov/34237169/. 34237169 10.1002/ajcp.12530

[r4] Tandon R. The historical roots and contemporary tendencies in participatory research: Implications for health care. De Koning K, Martin M, eds. Participatory Research in Health: Issues and Experiences. Zed Books; 1996:21–26. Accessed December 2025. https://pmc.ncbi.nlm.nih.gov/articles/PMC2566051/#ref1.

[r5] Robert Wood Johnson Foundation. Global ideas: Lessons on community power. Published June 2, 2022. Accessed December 2025. https://www.rwjf.org/en/about-rwjf/how-we-work/learning-and-evaluation/learning-across-global-borders/global-ideas-lessons-on-community-power.html#0.

[r6] Metre K, Paul N. Chinmaya Seva: CORD and Chinmaya Mission Hospital. Chinmaya Mission West; 2014.

[r7] Health for All Washtenaw. Adults without health insurance. Updated October 06, 2024. Accessed December 2025. https://www.healthforallwashtenaw.org/indicators/index/view?indicatorId=90&localeId=1363.

[r8] Washtenaw County Health Department. Maternal and infant health report. 2025. Accessed December 2025. https://content.civicplus.com/api/assets/1eedb74f-1332-40d2-a8b4-237632789028.

[r9] Cadez‑Martin A, Tan B Fox S, Matusko N, Gadepalli S. Effects of social determinants of health on infant mortality in Washtenaw and Wayne County, Michigan. Undergrad J Public Health. 2022;6:69–78. doi:10.3998/ujph.2313.

[r10] Bedaso A, Adams J, Peng W, Sibbritt D. The relationship between social support and mental health problems during pregnancy: A systematic review and meta‑analysis. Reprod Health. 2021;18(1):162. Accessed December 2025. https://link.springer.com/article/10.1186/s12978-021-01209-5. 34321040 10.1186/s12978-021-01209-5 PMC8320195

[r11] Lu MC, Johnson KA. Toward a national strategy on infant mortality. Am J Public Health. 2014;104(suppl 1):S13–S16. Accessed December 2025. https://ajph.aphapublications.org/doi/full/10.2105/AJPH.2013.301855. 24410337 10.2105/AJPH.2013.301855 PMC4011120

[r12] Ogbolu Y, Dudding R, Fiori K, et al. Global learning for health equity: A literature review. Ann Glob Health. 2022;88(1):89. Accessed December 2025. https://pmc.ncbi.nlm.nih.gov/articles/PMC9585985/. 36348705 10.5334/aogh.3810 PMC9585985

[r13] Kalendera U, Wiegmannb S, Ernstb M, Ihmeb L, Neumannb U, Stöckigt B. Who is sensitising whom? A participatory interview guide development as an awareness tool within a health care research project. Heliyon. 2023;9(6):e16778. doi:10.1016/j.heliyon.2023.e16778. 37484285 PMC10360575

[r14] Yancey L, LeBouef S. Parent education through conversation: The perceived effectiveness of parenting cafés in Southeast Missouri. National Council on Family Relations. 2020. Accessed December 2025. https://www.ncfr.org/ncfr-2020/session/425-208-ee-parent-education-through-conversation-perceived-effectiveness.

